# Migration–Gravity Sedimentation: An Effective Approach for Reducing Sperm DNA Fragmentation With Comparable Intracytoplasmic Sperm Injection Outcomes Compared to Density Gradient Centrifugation

**DOI:** 10.1002/rmb2.12680

**Published:** 2025-10-14

**Authors:** Hideaki Yajima, Hiroki Takeuchi, Kanako Kishi, Kazuki Yamagami, Akane Kagohashi, Erina Takayama, Masahide Shiotani, Noritoshi Enatsu, Eiji Kondo

**Affiliations:** ^1^ Department of Obstetrics and Gynecology, Graduate School of Medicine Mie University Tsu Japan; ^2^ Center of Advanced Reproductive Medicine Mie University Hospital Tsu Japan; ^3^ Department of Obstetrics and Gynecology Mie University Hospital Tsu Japan; ^4^ Hanabusa Women's Clinic Kobe Japan; ^5^ Hanabusa Men's Clinic Kobe Japan

**Keywords:** density gradient, DNA fragmentation, intracytoplasmic sperm injection, sperm motility, sperm selection

## Abstract

**Purpose:**

To compare the efficacy of migration–gravity sedimentation (MGS) and density gradient centrifugation (DGC) for sperm preparation in intracytoplasmic sperm injection (ICSI) cycles, focusing on sperm DNA fragmentation (SDF) and ICSI outcomes.

**Methods:**

In this prospective study, 32 patients who underwent ICSI using sibling oocytes were enrolled. Half of the oocytes were fertilized with DGC‐prepared sperm and the other half with MGS‐prepared sperm. Semen parameters were assessed using computer‐assisted sperm analysis (CASA), and SDF levels were measured using the terminal deoxynucleotidyl transferase dUTP nick end labeling assay before and after sperm preparation. Fertilization, blastocyst development, and clinical pregnancy rates were compared between the two groups.

**Results:**

MGS significantly reduced SDF levels compared to raw semen and DGC. CASA demonstrated enhanced motility, straightness, and linearity with MGS, although curvilinear velocity, average path velocity, and amplitude of lateral head displacement were lower. Fertilization and clinical outcomes, including blastocyst formation and pregnancy rates, were comparable between the groups.

**Conclusion:**

MGS is a simple, centrifuge‐free, and low‐cost sperm preparation technique that effectively reduces sperm DNA fragmentation and achieves ICSI outcomes similar to those of DGC. These findings indicate that MGS may be a viable alternative to assisted reproductive technology, specifically in patients without male‐factor infertility.

**Trial Registration:**

This study was registered in the University Hospital Medical Information Network (UMIN000043585)

## 1 Introduction

Infertility is defined as the inability of a healthy heterosexual couple to achieve a clinical pregnancy after 12 months of regular unprotected intercourse [1]. However, for women aged ≥ 35 years, infertility treatment is recommended after 6 months, and for those aged > 40 years, it should begin immediately [2]. The prevalence of infertility varies by region but is reportedly between 12.6% and 17.5% [3], with rates expected to rise owing to lifestyle changes, such as delayed marriage and childbearing [4]. Approximately 20%–30% of infertility cases are attributed solely to male factors, and male infertility accounts for approximately 50% of all cases [5]. Therefore, a thorough assessment of male infertility is crucial. Male infertility can result from hormonal and anatomical abnormalities, genetic factors [6], lifestyle and environmental factors [7], and oxidative stress [8]. Semen analysis is the standard initial screening test used in clinical practice. Although the World Health Organization provides reference values for semen parameters [9], these are based on men whose partners conceived naturally within a year and are not predictive of assisted reproductive technology outcomes or pregnancy rates.

Sperm DNA fragmentation (SDF) has recently emerged as a marker for sperm quality. It involves the breakage of one or both strands of sperm DNA caused by inadequate chromatin condensation owing to improper protamination, disrupted apoptosis, and oxidative stress [10]. High SDF levels are associated with lower natural pregnancy rates [11], increased miscarriage rates [12, 13], recurrent pregnancy loss, and implantation failure [14, 15], reduced live birth rates [16], and reduced in vitro fertilization (IVF) success rates [12, 17]. Therefore, SDF is a primary concern in fertility treatment.

In IVF, standard sperm selection techniques include density gradient centrifugation (DGC) [18], the swim‐up method [19], or a combination of both methods [20]. Although these methods significantly reduce SDF levels, they may paradoxically increase SDF levels in certain cases [21]. Recently, alternative centrifuge‐free methods, such as migration–gravity sedimentation (MGS) [22] and microfluidic sperm sorting (MSS) [23, 24] have been introduced. Although MSS has demonstrated promise in enhancing IVF outcomes, it is expensive, and its benefits are mostly limited to enhancing fertilization per mature oocyte when used indiscriminately, highlighting the need for careful patient selection [25]. In contrast, MGS is an older, low‐cost technique that leverages sperm motility and gravity to isolate functionally competent sperm without centrifugation [26, 27]. Although its efficacy in intrauterine insemination has recently been reported [28], evidence of its utility in IVF remains limited.

In this study, we prospectively assessed patients who underwent intracytoplasmic sperm injection (ICSI) using sperm prepared by either DGC or MGS from sibling oocytes retrieved during the same cycle. We assessed the semen parameters, SDF levels before and after sperm preparation, and ICSI outcomes.

## 2 Materials and Methods

### 2.1 Ethical Approval

This study included patients who underwent IVF, provided informed consent, and met the eligibility criteria at Mie University Hospital and Hanabusa Women's Clinic between April 2021 and March 2024. Male participants were required to be at least 20 years old, and female participants were required to be between 20 and 50 years old, with the potential to yield multiple oocytes. Exclusion criteria included a total motile sperm count of < 20 × 10^6^ cells, use of frozen or testicular sperm, or a diagnosis of varicocele. Data were collected using a questionnaire on the abstinence period, smoking history, and alcohol consumption of the male participants. This study was approved by the Clinical Research Ethics Review Committee of Mie University Hospital (H2020‐252) and adhered to the guidelines of the Ethics Committee of the Japan Society of Obstetrics and Gynecology (No. 116). Additionally, the study was registered with the University Hospital Medical Information Network (UMIN000043585).

### 2.2 Study Design

This study was designed as a pilot investigation to explore the potential efficacy and feasibility of MGS compared to DGC in terms of SDF and ICSI outcomes. The target sample size was pragmatically set based on patient availability over a fixed study period, aiming to gather preliminary data to inform the design of a future adequately powered study. When multiple oocytes were retrieved, sibling oocytes were fertilized through ICSI using sperm prepared with either the DGC or MGS method. Sperm motility and related parameters were assessed using computer‐assisted sperm analysis (CASA). SDF values were measured in sperm samples discarded after ICSI.

### 2.3 Treatment Protocol

Ovarian stimulation protocols and gonadotropin dosages were determined based on the patient's ovarian reserve, and all patients underwent ICSI. The natural cycle protocol involved the administration of clomiphene citrate or letrozole from the early follicular phase, with gonadotropins added as necessary. In the gonadotropin‐releasing hormone (GnRH) agonist long protocol, buserelin acetate nasal spray was initiated on day 21 of the previous menstrual cycle. In the GnRH agonist short protocol, buserelin was initiated on day 2 of the cycle, along with gonadotropin administration from the early follicular phase. In the GnRH antagonist protocol, gonadotropins were initiated from the beginning of the cycle, and either 0.25 mg cetrorelix acetate or ganirelix acetate was added once the dominant follicle reached 14–15 mm in diameter. When the leading follicle reached 17–18 mm, ovulation was triggered using either 250 μg of chorionic gonadotropin alpha, 5000 IU of human chorionic gonadotropin (hCG), or buserelin acetate nasal spray, followed by oocyte retrieval 36 h later. The oocytes were denuded using hyaluronidase, and their maturity was confirmed. Fertilization was checked the day after ICSI, and the embryos were cultured until the blastocyst stage. Blastocysts were assessed using the Gardner grading system, and those graded as BL3BB or higher were vitrified. Embryo transfer was conducted using either a hormone replacement or natural cycle protocol, based on the patient's ovulatory status. Endometrial thickness was confirmed to be at least 8 mm at the time of transfer. All patients underwent single embryo transfer. On the day of transfer, embryos were thawed, cultured in recovery medium (Kitazato, Tokyo, Japan), and transferred using a vaginal catheter (Kitazato, Tokyo, Japan). Serum hCG values were measured 14 days post‐transfer to assess pregnancy. Clinical pregnancy was confirmed by the presence of a gestational sac on ultrasound, and patients were referred to their preferred obstetric facility at approximately the 9th week of gestation.

### 2.4 Semen Analysis

Liquefied semen samples were analyzed using the Sperm Motility Analysis System (SMAS; Ditect, Tokyo, Japan)—a CASA system. The parameters measured by SMAS included total sperm concentration, motility rate, straight‐line velocity (VSL), curvilinear velocity (VCL), average path velocity (VAP), straightness (STR), linearity (LIN), amplitude of lateral head displacement (ALH), and beat cross frequency.

### 2.5 Sperm Preparation

Semen samples were collected by masturbation and analyzed after liquefaction. For the DGC method, semen was layered over 1 mL of 40% PureCeption and 2 mL of 80% PureCeption (Cooper Surgical, CT, USA), followed by centrifugation (200× *g*, 10 min). The supernatant was discarded, and the pellet was resuspended in 1 mL of Universal IVF Medium and centrifuged again (200× *g*, 5 min). The final sperm suspension was prepared in 0.1 mL of Universal IVF Medium and analyzed using SMAS.

**FIGURE 1:**
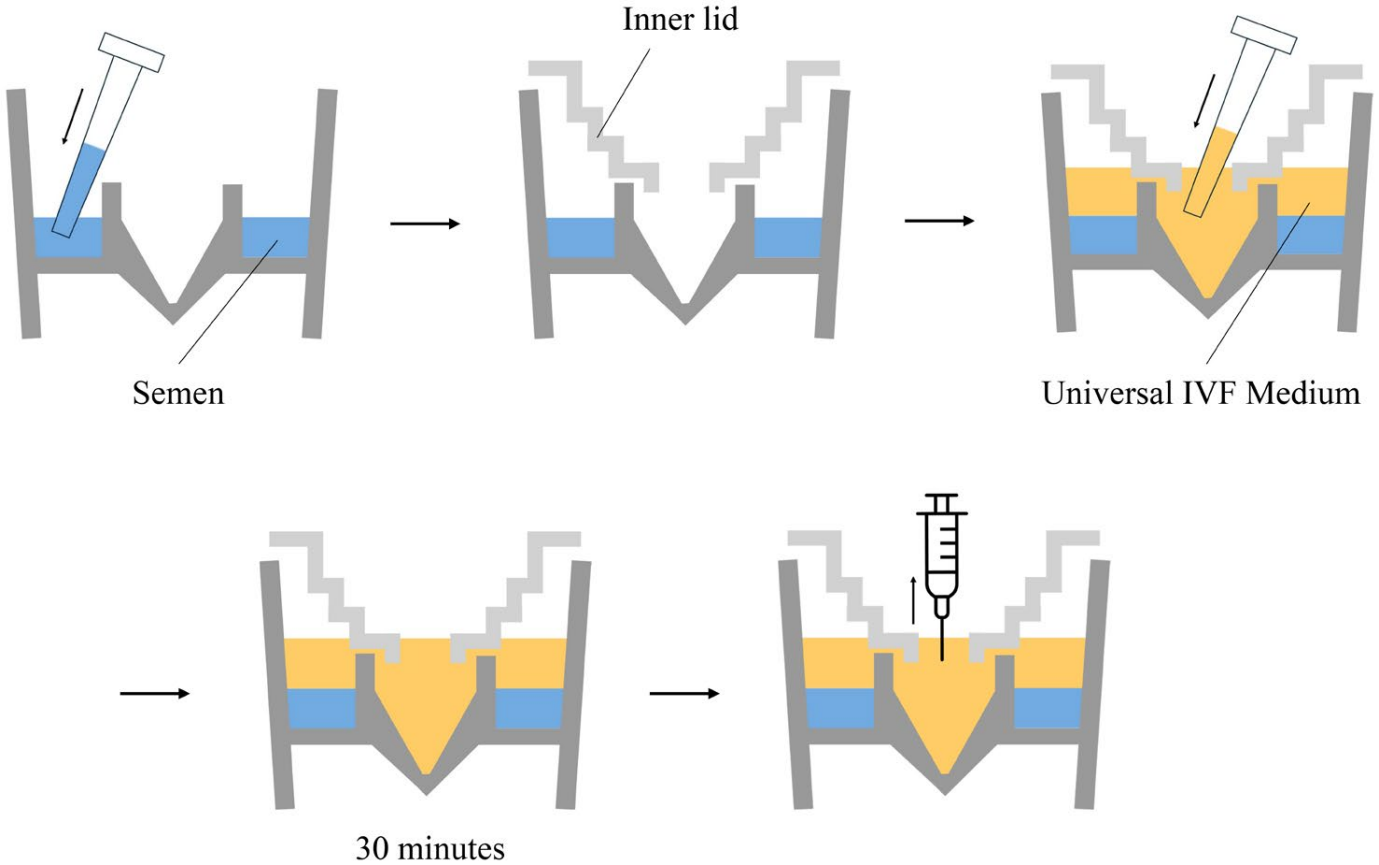
Schematic representation of the migration–gravity sedimentation (MGS) method. Motile sperm migrate into the inner tube filled with culture medium by gravity.

The MGS method was performed using MIGLIS (Menicon Life Science, Japan) with a partially modified version of the manufacturer's protocol (Figure 1). Semen was added to the injection chamber of the main body, and the inner lid was secured in place. Then, 2 mL of Universal IVF Medium was added to the inner tube of the main body. After attaching the outer lid, the device was left to stand for 30 min to allow motile sperm to be separated by gravity. After 30 min, 0.4 mL of the sperm suspension was carefully collected from the bottom of the inner tube using a 1 mL tuberculin syringe (BD, NJ, USA).

### 2.6 SDF Level Measurement

For both raw semen and sperm processed by each method, 1 × 10^6^ cells were collected and fixed with 4% paraformaldehyde (Nacalai Tesque, Kyoto, Japan) at 4°C for 30 min. Subsequently, the samples were centrifuged at room temperature, and the supernatant was discarded. The mixture was washed by adding 1 mL of phosphate‐buffered saline (Nacalai Tesque, Kyoto, Japan), followed by centrifugation. This washing step was repeated twice. Subsequently, the samples were permeabilized by adding 1 mL of 70% ethanol (Muto Pure Chemicals, Tokyo, Japan) and stored at −20°C to −30°C. To detect SDF, the terminal deoxynucleotidyl transferase dUTP nick end labeling (TUNEL) assay was performed using the BD Pharmingen APO‐DIRECT Kit (BD Biosciences), following the manufacturer's protocol. After permeabilization, the sperm were washed and incubated at 37°C for 1 h in a staining solution containing terminal deoxynucleotidyl transferase (TdT) and fluorescein‐dUTP (dUTP‐fluorescein isothiocyanate [FITC]). A negative control solution lacking TdT was used to confirm staining specificity. This process enabled the fluorescent labeling of DNA breaks using FITC. Following incubation, the sperm were washed with a rinse buffer to remove excess stain. Finally, propidium iodide (PI)/RNase was added to counterstain the sperm, allowing differentiation between fragmented and non‐fragmented DNA. Flow cytometric analysis was performed using a MACSQuant Analyzer 16 and MACSQuantify software (Miltenyi Biotec, North Rhine‐Westphalia, Germany). Doublets were excluded, and gating was performed based on forward and side scatter. PI‐positive sperm were gated, and the FITC fluorescence threshold was determined using the negative control. Sperm exhibiting FITC fluorescence above the threshold were considered FITC‐positive. The percentage of FITC‐positive sperm among the PI‐positive population was calculated as the SDF level. In all measurements, a minimum of 5000 PI‐positive sperm were analyzed.

### 2.7 Statistical Analysis

Statistical analyses were performed using GraphPad Prism version 10 (GraphPad Software, CA, USA). For SDF values and sperm parameters measured using CASA, the normality of data distribution was assessed using the Shapiro–Wilk test. Variables not following a normal distribution were analyzed using the Friedman test, with post hoc pairwise comparisons performed by Dunn's multiple comparisons test when significance was detected (*p* < 0.05). Normally distributed variables were analyzed using repeated‐measures ANOVA; if Mauchly's test of sphericity was violated, the Greenhouse–Geisser correction was applied, followed by Bonferroni‐adjusted multiple comparisons when appropriate. ICSI outcomes between the DGC and MGS groups were compared using Fisher's exact test. Statistical significance was set at *p* < 0.05.

## 3 Results

### 3.1 Case Selection Flowchart and Patient Characteristics

**FIGURE 2:**
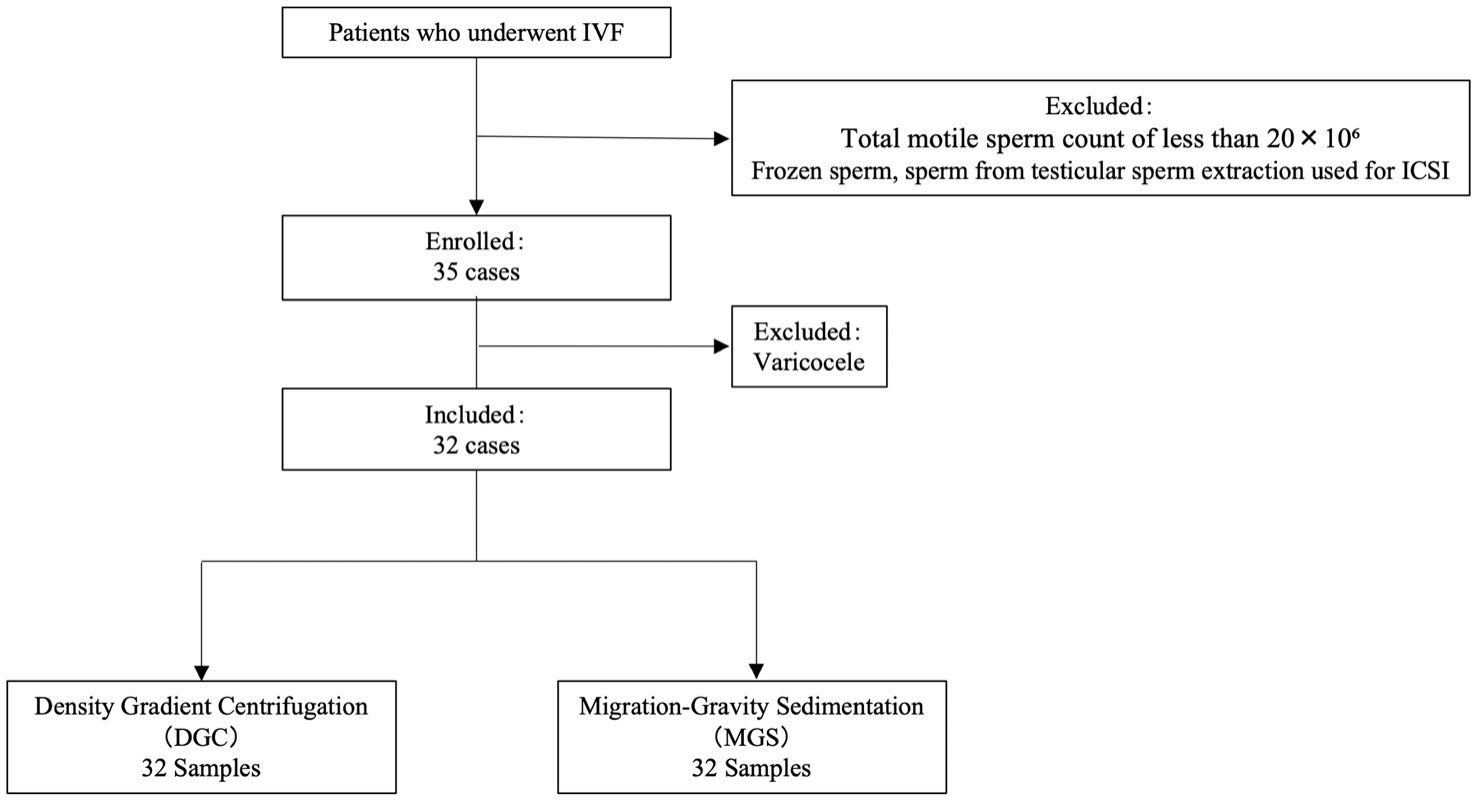
Case selection flowchart. A total of 35 couples were enrolled in this study. Three patients were excluded because of the presence of varicocele. The remaining 32 couples proceeded with the study protocol, in which sibling oocytes retrieved from a single cycle were fertilized by intracytoplasmic sperm injection using sperm prepared by either density gradient centrifugation or migration–gravity sedimentation. All cases were included in the final analyses of semen parameters, sperm DNA fragmentation, and in vitro fertilization outcomes.

A total of 35 patients were enrolled in this study, of which three were excluded because of varicocele (Figure 2). The background characteristics of the remaining 32 couples are presented in Table 1. The median age of the male partners was 36 years, with a body mass index (BMI) of 22.9 and an abstinence period of 2 days. Among them, 5 were smokers and 9 consumed alcohol. The median age of the female partners was 35 years—9 had no prior pregnancies and 23 had a history of pregnancy. Twenty‐one had no history of childbirth, whereas 11 had given birth once or twice. The median BMI was 20.8, and the median anti‐Müllerian hormone level was 3.56 ng/mL. The most common infertility factor was tubal (*n* = 24), followed by unexplained infertility (*n* = 5), uterine (*n* = 3), male (*n* = 3), and ovarian factors (*n* = 1), with certain cases involving multiple factors. Ovarian stimulation protocols were selected based on each patient's ovarian reserve that included natural cycle (*n* = 23), GnRH antagonist (*n* = 6), GnRH agonist long (*n* = 2), and GnRH agonist short protocols (*n* = 1).

**TABLE 1:** Patient characteristics.

*Male partner*	
Age (years)	36 (28–49)
BMI (kg/m^2^)	22.9 (17.7–27.7)
Abstinence period (day)	2 (0–6)
Smoking	5
Alcohol	9
*Female partner*	
Age (years)	35 (27–44)
Gravidity	
0	9
1	9
2	7
3	1
> 4	6
Parity	
0	21
1	10
2	1
> 3	0
BMI (kg/m^2^)	20.8 (16.8–25.7)
AMH (ng/mL)	3.56 (0.64–17.31)
Infertility factor	
Uterine	3
Tubal	24
Ovarian	1
Male	3
Unexplained	5
Ovarian stimulation	
Natural	23
Antagonist	6
Long	2
Short	1

Abbreviations: AMH, anti‐Mullerian hormone; BMI, body mass index.

### 3.2 Sperm Parameter

Sperm retrieved from each preparation method of the 32 male participants was analyzed using the SMAS system (Table 2). Compared to the raw semen (RAW group), the MGS group exhibited significantly higher motility, VSL, VCL, VAP, and ALH values, whereas the sperm concentration and STR values were significantly lower. Additionally, compared to the DGC group, the MGS group had significantly higher motility and STR values and significantly lower sperm concentration and ALH values.

**TABLE 2:** Comparative analysis of semen parameters between RAW, density gradient centrifugation, and migration–gravity sedimentation groups.

	RAW	DGC	MGS
Sperm volume (mL)	3.0 (2.0–5.5)	N/A	N/A
Concentration (×10^6^/mL)	74.0 (14.48–218.5)^a,b^	14.04 (4.78–46.53)^a,c^	2.18 (0.22–9.20)^b,c^
Total motility (%)	43.11 (16.23–79.42)^a,b^	73.80 (10.54–90.77)^a,c^	86.18 (38.89–100.0)^b,c^
VSL (μm/s)	23.19 (13.18–37.46)^a,b^	30.84 (17.18–67.53)^a^	32.21 (13.90–49.89)^b^
VCL (μm/s)	53.13 (26.30–82.37)^a,b^	105.1 (80.65–143.2)^a,c^	89.0 (64.0–138.6)^b,c^
VAP (μm/s)	36.39 (21.07–54.05)^a,b^	54.05 (44.18–76.81)^a,d^	49.81 (32.41–72.96)^b,d^
STR (%)	0.435 (0.32–0.64)^a,d^	0.33 (0.20–0.62)^a,b^	0.395 (0.21–0.54)^b,d^
LIN (%)	0.625 (0.47–0.79)	0.625 (0.37–0.85)^d^	0.695 (0.39–0.85)^d^
ALH (μm)	1.2 (0.49–1.83)^a,b^	2.285 (1.76–3.33)^a,c^	1.905 (1.33–3.09)^b,c^
BCF (Hz)	8.97 (7.86–11.75)	9.385 (7.59–16.32)	9.585 (6.87–13.89)

*Note:* Superscripts: ^a,b,c^ *p* < 0.01; ^d^ *p* < 0.05.

Abbreviations: ALH, amplitude of lateral head displacement; BCF, beat cross frequency; DGC, density gradient centrifugation; LIN, linearity; MGS, migration‐gravity sedimentation; N/A, not available; RAW, raw semen; STR, straightness; VAP, average path velocity; VCL, curvilinear velocity; VSL, straight‐line velocity.

### 3.3 Comparison Between the Three Groups Based on SDF Values

**FIGURE 3:**
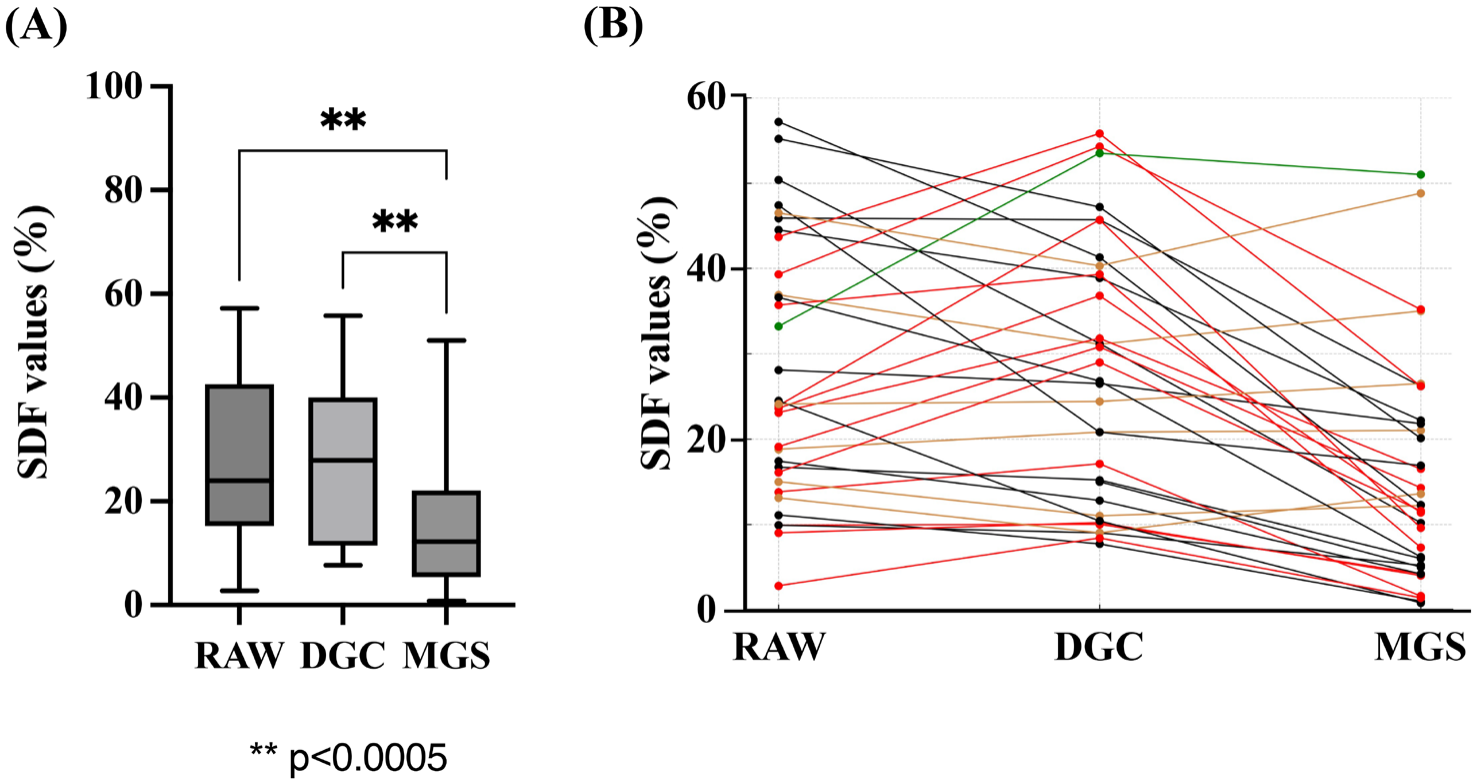
Comparison of sperm DNA fragmentation (SDF) levels among the RAW, density gradient centrifugation (DGC), and migration–gravity sedimentation (MGS) groups. (A) Box plots demonstrating SDF levels, measured using the terminal deoxynucleotidyl transferase dUTP nick end labeling assay and flow cytometry in raw semen (RAW) and sperm processed by DGC and MGS. (B) Lines represent individual patient transitions in SDF values across the RAW, DGC, and MGS groups. Red lines indicate cases in which SDF values increased after DGC compared to RAW. Green lines indicate cases in which SDF values increased after MGS compared to RAW. Ochre (Peru) lines indicate cases in which SDF values were higher after MGS than after DGC. Black lines represent cases that did not meet any of the above conditions.

SDF values were measured using flow cytometry following TUNEL staining and compared among original semen and the two sperm preparation methods (Figure 3). No significant differences were observed between the RAW and DGC groups. However, SDF values in the MGS group were significantly lower than those in the RAW and DGC groups (Figure 3A). SDF values increased after DGC compared to that of raw semen in 15 cases. In contrast, both DGC and MGS resulted in lower SDF values than raw semen in a total of 15 cases. Similarly, SDF values increased after MGS in 5 cases. In 6 cases, SDF values after MGS were higher than those after DGC (Figure 3B).

### 3.4 ICSI Outcome

IVF was performed in 32 female partners (Table 3). In total, 139 and 151 mature oocytes were used for ICSI in the DGC and MGS groups, respectively. Fertilization rates were 84.9% and 90.7% for the DGC and MGS groups, respectively. Cleavage stage development rates were 63.3% and 68.9%, and blastocyst formation rates were 21.6% and 21.9%, respectively. No significant differences were observed between the groups. Embryo transfers were performed in 9 (28.1%) and 13 (40.6%) cases in the DGC and MGS groups, respectively. Among these, hormone replacement cycle frozen embryo transfers (FETs) were performed in 6 (60.0%) and 9 (56.3%) cases in the DGC and MGS groups, respectively, whereas natural cycle FETs were performed in 4 (40.0%) and 7 (43.8%) cases, respectively. No significant differences were observed between the groups. The implantation rate, clinical pregnancy, miscarriage per transfer, and live birth rates were not significantly different between the groups.

**TABLE 3:** Ovarian stimulation, implantation, and clinical pregnancy outcomes.

	DGC	MGS	*p*
*Ovarian stimulation outcomes*			
No. of patients	32	32	
Number of MII oocytes	139	151	
Fertilization rate	118/139 (84.9%)	137/151 (90.7%)	ns
Cleavage‐stage embryo formation rate	88/139 (63.3%)	104/151 (68.9%)	ns
Blastocyst formation rate	30/139 (21.6%)	33/151 (21.9%)	ns
*Implantation and clinical pregnancy outcomes*			
No. of patients	9	13	
No. of blastocyst transfer cycle	10	16	
Hormone replacement cycle frozen embryo transfer	6/10 (60.0%)	9/16 (56.3%)	ns
Natural cycle frozen embryo transfer	4/10 (40.0%)	7/16 (43.8%)	ns
Implantation rate per embryo transfer	8/10 (80.0%)	11/16 (68.8%)	ns
Pregnancy rate per embryo transfer	7/10 (70.0%)	7/16 (43.8%)	ns
Miscarriage rate	1/10 (10.0%)	0/16 (0.0%)	ns
Live birth rate	5/9 (55.6%)	6/13 (46.2%)	ns

Abbreviations: DGC, density gradient centrifugation; MGS, migration‐gravity sedimentation; ns, not significant.

## 4 Discussion

This study aimed to assess whether MGS can serve as an alternative to DGC by comparing sperm motility parameters, SDF levels, and IVF outcomes. We observed that MGS, which collects motile sperm without centrifugation, resulted in lower SDF levels than DGC. Additionally, the analysis of sperm motility parameters using CASA indicated enhancements in several motility parameters with MGS. These findings indicate that MGS may be a valuable alternative to DGC for reducing sperm damage more effectively. In contrast, fertilization, blastocyst cryopreservation, implantation, clinical pregnancy, and live birth rates were comparable between the two methods. To the best of our knowledge, this is the first prospective study to compare ICSI outcomes using MGS‐ and DGC‐selected sperm in patients without male factor infertility.

According to the CASA‐based analysis of sperm motility parameters, MGS enhanced the motility rate, STR, and LIN of sperm compared to that of DGC, whereas their VCL, VAP, and ALH levels were lower. Semen is composed of spermatozoa and seminal plasma, which contain various energy substrates, such as fructose, ions, and amino acids [29]. These components support sustained progressive motility through efficient ATP production [30]. In contrast, itaconic acid regulates anaerobic glycolysis by suppressing tail amplitude [31]. DGC physically removes seminal plasma, bacteria, leukocytes, non‐sperm cells, and dead sperm. Sperm processed by DGC exhibit higher VCL and ALH but lower LIN [32]. These findings indicate that the removal of seminal plasma by DGC may enhance anaerobic glycolysis, resulting in increased VCL, VAP, and ALH while reducing STR. Meanwhile, owing to the structural characteristics of the MGS—where motile sperm migrate into a central tube filled with culture medium after semen injection—seminal plasma may not be completely removed. As a result, STR and LIN were higher, whereas VCL, VAP, and ALH were lower in the MGS group than in the DGC group.

In this study, the SDF levels were not significantly different between the raw semen and DGC groups, whereas the MGS group demonstrated the lowest values. Although DGC is the most commonly used technique in assisted reproductive technology, centrifugation may cause mechanical damage to sperm, potentially increasing SDF levels [24]. One possible explanation is that when sperm are processed on a discontinuous colloidal silica gradient, certain metals in the medium may facilitate free radical generation, resulting in oxidative DNA damage [33]. In our study, SDF levels increased after DGC in 15 patients (46.9%), which may explain the absence of a significant difference between the raw semen and DGC groups. In contrast, MGS selects sperm based on motility [34]. Sperm processed with MGS have higher viability and motility, lower rates of mitochondrial damage, and lower SDF levels than those processed with DGC [22]. Our findings were consistent with these reports and indicate that nearly half of the patients exhibited increased SDF after DGC, whereas this was less frequently observed following MGS. Sperm with lower levels of DNA fragmentation generally maintain higher motility [8, 11], and MGS—by relying on motility‐driven gravity sedimentation—preferentially enriches such sperm. Thus, the superior SDF profile in the MGS group can be largely explained by the preferential recovery of highly motile sperm, which are more likely to have intact DNA. At the same time, centrifugation may independently contribute to DNA damage through mechanical stress and ROS generation, as suggested in previous studies [33]. We therefore consider that both factors act in concert to improve SDF outcomes in the MGS group. Nevertheless, a small subset of cases (15.2%) showed increased SDF even after MGS, indicating that this method is not completely protective. One possible explanation is that motile but morphologically abnormal sperm might have been included in some MGS‐processed samples, which could have contributed to the increased SDF values observed in these cases.

No significant differences in ICSI outcomes were observed between the DGC and MGS groups. Similar to MGS, the swim‐up technique is a commonly used centrifuge‐free method for sperm preparation. Previous studies comparing DGC and swim‐up in IVF have reported similar fertilization, embryo quality, blastocyst formation, and live birth rates per transfer, even in ICSI cases [35]. Although it was anticipated that reducing SDF levels through centrifuge‐free sperm selection may enhance IVF outcomes, no enhancement was observed. One possible explanation is that all fertilizations in this study were performed using ICSI. High SDF levels are not associated with lower pregnancy or live birth rates during ICSI cycles [36, 37]. Because ICSI involves the direct injection of morphologically selected sperm into the oocyte, the effect of DNA damage may be mitigated. Moreover, although no statistically significant differences were observed, the MGS group showed slightly lower outcomes in terms of implantation, pregnancy, and live birth rates compared to the DGC group. This may be attributed to the relatively small number of embryo transfers in both groups, which may have limited the statistical power to detect significant differences. Additionally, factors such as endometrial condition, hormonal environment, and individual patient preferences may have influenced the timing and outcomes of embryo transfer, regardless of the sperm preparation method.

This study has several limitations. Because MGS requires a certain motile sperm count, patients with severe male factor infertility and those using frozen sperm were excluded. Additionally, sperm motility parameters were assessed using the SMAS; however, sperm morphology could not be assessed. Finally, as previously noted, the limited number of cases may have restricted the statistical power to fully evaluate differences in ICSI outcomes.

In conclusion, MGS is a relatively low‐cost, centrifuge‐free technique that reduces SDF and yields ICSI outcomes comparable to those of DGC. Compared to DGC, MGS is simpler to perform and is associated with fewer cases of increased SDF, selecting sperm with lower levels of DNA damage. Further studies with larger sample sizes are required to clarify the relationships between sperm parameters, SDF levels, and ICSI outcomes.

## Ethics Statement

All procedures followed the ethical standards of the institutional and national committees on human experimentation and the Helsinki Declaration (1964) and its later amendments. Informed consent was obtained from all patients. The study was approved by the Clinical Research Ethics Review Committee of Mie University Hospital (H2020‐252) and adhered to the guidelines of the Ethics Committee of the Japan Society of Obstetrics and Gynecology (No. 116).

## Conflicts of Interest

H. Takeuchi has received a joint research grant from Menicon Co. Ltd. The other authors declare no conflicts of interest associated with this manuscript.

## Data Availability

The data supporting the findings of this study is available from the corresponding author, Hiroki Takeuchi, upon request.
